# Supplementary material for: Temporal demands of elite fencing
Source: PLoS One. 2023 Jun 1;18(6):e0285033. doi: 10.1371/journal.pone.0285033 (PMC10234521; doi:10.1371/journal.pone.0285033)
Supplement: Attachment — Submitted filename: Rebuttal Letter.docx [file pone.0285033.s001.docx]

Response to Reviewer Comments

Thank you for reviewing our paper. We have gone through the feedback and tried to make the amendments suggested. Hopefully this has improved the manuscript.

**Reviewer1:** Reviewer #1: The subject of the study is very interesting. I am very pleased that these kinds of research investigations are also being created. In my opinion, thanks to such studies we can better understand the load of athletes in fencing. These findings can be applied within the training environment and performance enhancement. I also believe that the analysis of temporal relationships within weapons will help other potential researchers to follow in detail, for example, the physiological aspects of the load during matches or training. Adjusting training according to the data obtained can ensure performance growth. The authors rightly point out that current knowledge in this issue is based on research information that is "relatively" old. It is necessary to mention that today's fencing is different from the fencing that was practiced 30 years ago. It can be argued that fencing has definitely gotten faster.

**Response:** Thank you for the positive comments about our paper.

**Reviewer 1:** The title of the manuscript is too specific, focused on one part of the solved problem. Wouldn't it be better to introduce Analysis of the Time Interactions in Elite Fencing?

**Response:** Thank you for the suggestion, we have now amended the title to ‘Temporal demands of elite fencing’ to reflect this.

**Reviewer 1**: In my opinion, words should not be repeated in the article title and keywords.

**Response:** We have now changed the keywords to be different to those in the title.

**Reviewer 1:** The abstract is apt. The only thing I would modify: it is not clear what the average working time and work-to-rest ratios are.

**Response:** We have provided a definition of work time which hopefully improves the clarity.

**Reviewer 1**: The results in Abstract are detailed, but there is no explanation of the time intervals. Maybe it should generalized in this part. Perhaps at the end of the abstract I would emphasize where the results can be applied.

**Response**: Thank you for the comment. Hopefully the explanation of work time helps clarify this. As much as we agree with you that it would be good to emphasise how the results can be applied, we are constrained by the abstract word count.

**Reviewer 2:** Please, improve in the discussion section the relationship between time analysis and fencing energetics. Can we propose different energetic mechanisms for each weapon? No more than a paragraph, please.

**Response**: We have added an additional paragraph starting line 273 on page 16 which hopefully expands on this.
